# Gambogic Acid Lysinate Induces Apoptosis in Breast Cancer MCF-7 Cells by Increasing Reactive Oxygen Species

**DOI:** 10.1155/2015/842091

**Published:** 2015-03-18

**Authors:** Yong-Zhan Zhen, Ya-Jun Lin, Kai-Ji Li, Xiao-Shan Yang, Yu-Fang Zhao, Jie Wei, Jing-Bo Wei, Gang Hu

**Affiliations:** ^1^Department of Histology and Embryology, Basic Medical College of Hebei United University, Tangshan 063000, China; ^2^The Key Laboratory of Geriatrics, Beijing Hospital & Beijing Institute of Geriatrics, Ministry of Health, No. 1 Dahua Road, Dongdan, Dongcheng District, Beijing 100730, China

## Abstract

Gambogic acid (GA) inhibits the proliferation of various human cancer cells. However, because of its water insolubility, the antitumor efficacy of GA is limited.* Objectives.* To investigate the antitumor activity of gambogic acid lysinate (GAL) and its mechanism.* Methods.* Inhibition of cell proliferation was determined by MTT assay; intracellular ROS level was detected by staining cells with DCFH-DA; cell apoptosis was determined by flow cytometer and the mechanism of GAL was investigated by Western blot.* Results.* GAL inhibited the proliferation of MCF-7 cells with IC_50_ values 1.46 *μ*mol/L comparable with GA (IC_50_, 1.16 *μ*mol/L). GAL promoted the production of ROS; however NAC could remove ROS and block the effect of GAL. GAL inhibited the expression of SIRT1 but increased the phosphorylation of FOXO3a and the expression of p27Kip1. At knockdown of FOXO3a, cell apoptosis induced by GAL can be partly blocked. In addition it also enhanced the cleavage of caspase-3.* Conclusions.* GAL inhibited MCF-7 cell proliferation and induced MCF-7 cell apoptosis by increasing ROS level which could induce cell apoptosis by both SIRT1/FOXO3a/p27Kip1 and caspase-3 signal pathway. These results suggested that GAL might be useful as a modulation agent in cancer chemotherapy.

## 1. Introduction

Breast cancer represents the most common cancer in women in western countries. One in 8 women in the United States will develop breast cancer in her lifetime, while its incidence rate constantly increases in developing countries [1, 2]. Over the past decade, although the introduction of new drugs, including paclitaxel and Herceptin, has improved the treatment landscape for breast cancer patients, some patients continue to experience drug resistance and disease relapse [3, 4]. Therefore, further investigations to find a novel anti-breast-cancer drug should be conducted.

Gambogic acid (GA) is the principal active component of gamboge, the resin from various* Garcinia* species including* G. Morella and G. hanburyi* [5]. It was reported in traditional Chinese medical documents that GA possessed diverse biological effects such as anti-inflammatory and antipsoriatic efficacy [6, 7], anti-invasive effect [8], inhibiting angiogenesis [9], and inducing cell apoptosis [10]. One of the major barriers for GA clinical application is its insolubility in water. GA could only dissolve in DMSO [11], and DMSO is rather harmful to cells. Even though it dissolves in DMSO, if diluted by water, it will precipitate. Thus it impedes the uses of GA. In the present study, we prepared gambogic acid lysinate (GAL), which can dissolve in water, and the solubility of GAL in water is 1.16 g. No report about the manufacture of GAL and its antitumor activity has been found yet. In this study, we investigated the antitumor activity of GAL.

## 2. Materials and Methods

### 2.1. Chemicals and Antibodies

Gambogic acid (98%) was purchased from Nanjing Jingzhu Bio-technology Ltd. (Nanjing, Jiangsu, China). Lysine was purchased from Beijing Solarbio Science and Technology Co. (Beijing, China). Gambogic acid lysinate (GAL) was made in our department. 3-(4,5-Dimethylthiazol-2-yl)-2,5-diphenyltetrazolium bromide (MTT) and dimethyl sulfoxide (DMSO) were obtained from Sigma Aldrich (Shanghai, China). FOXO3a siRNA and the scrambled siRNA (NC siRNA) control were supplied by Santa Cruz Technology (Dallas, TX, USA). Antibodies against SIRT1, FOXO3a, p-FOXO3a (s294), p27Kip1, caspase-3 and cleaved-caspase-3 (C-caspase-3) were purchased from Cell Signaling Technology (Beverly, MA, USA). Antibody against *β*-actin was purchased from Santa Cruz Technology (Dallas, TX, USA). Secondary antibodies were purchased from Cell Signaling Technology.

### 2.2. The Protocol of GAL Preparation and Matrix-Assisted Laser Desorption/Ionization Time of Flight Mass Spectrometry (MALDI-TOF MS) Analysis

**Figure 1:**
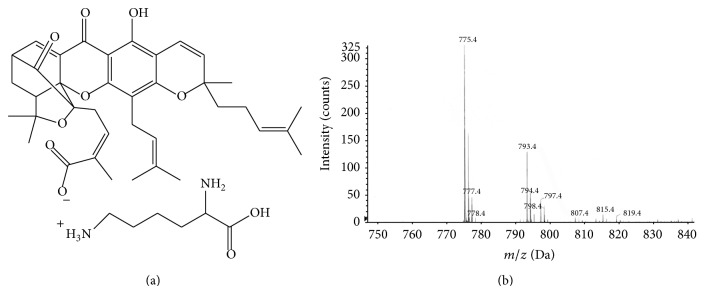
The structure and the determination of gambogic acid lysinate. Gambogic acid and lysine combine together through ionic bond. It is determined by Matrix-Assisted Laser Desorption/Ionization Time of Flight Mass Spectrometry and the peak value (755.4 Da) is gambogic acid lysinate.

Lysine (29.2 mg) was dissolved in distilled water and then gambogic acid (62.9 mg) was added to lysine solution. After lysine reacted with gambogic acid at 30°C for 24 hours, the solution was frozen and dried in freeze-drier (SCIENTZ, Ningbo, China) for 12 hours. An Agilent 6500 MS system (Agilent Technologies, CA, USA) was used to analyze the molecular weight of new product. The analysis method is following. Ionization is achieved using electrospray in the positive mode with the spray voltage set at 4.0 kV. The fragmentor voltage and collision energy were optimized during tuning as 150, 18 eV for GAL. Analysis was carried out in electrospray positive ionization using multiple reaction monitoring modes. The mass transition ion pair was selected as* m/z* 840→750 for GAL. The ion spectra of product are represented in Figure 1(b). The data acquisition software used was MassHunter software (Agilent Corporation, MA, USA).

### 2.3. Cell Culture and Cytotoxicity Assay

Human breast cancer cell lines MCF-7 were used in this study. MCF-7 cells were maintained in DMEM medium supplemented with 10% fetal bovine serum (Hyclone, Logan, UT, USA). Cells were grown in a humidified atmosphere of 5% CO_2_ at 37°C. In vitro cytotoxicity of GAL was determined using 3-(4,5-dimethylthiazol-2-yl)-2,5-diphenyltetrazolium bromide (MTT) assay. Briefly, MCF-7 cells were transferred to 96-well tissue culture plates at a density of 3 × 10^3^ cells per well, 24 hours prior to treatment. The medium was then replaced with fresh medium containing GAL at different concentrations. The culture medium without any drug formulation was used as the control. After 48 hours of incubation, medium was removed and cells were washed once with sterile phosphate buffered saline (PBS). Then 20 *μ*L of MTT solution (5 mg/mL) was added to each well and further incubated for 4 hours. Medium was removed and 150 *μ*L DMSO was added to each well to dissolve the purple formazan crystal converted from MTT. Optical density at 570 nm was determined with a SpectraMax 190 Absorbance Microplate Reader (Sunnyvale, CA, USA) and the concentration at which 50% of growth is inhibited (IC_50_) was calculated by GraphPad Prism 5.0 (GraphPad, La Jolla, CA, USA).

### 2.4. Examination of Intracellular Reactive Oxygen Species (ROS) Accumulation

Intracellular hydrogen peroxide levels were monitored by fluorescence microscopy and fluorescence spectrophotometer after staining with DCFH-DA (dichloro-dihydro-fluorescein diacetate; Molecular Probes, Eugene, OR, USA). Briefly, cells in a logarithmic growth phase (2 × 10^5^ cells per well in a 25 mm^2^ polystyrene culture flask) were treated with GAL for 24 h and and then labeled with 10 *μ*mol/L DCFH-DA for 1 h. Next, the cells were monitored by Olympus inverted fluorescence microscope (Tokyo, Japan) and fluorescence spectrophotometer (Cary Eclipse, Palo Alto, CA, USA). The percentage of cells displaying increased dye uptake was used to reflect an increase in ROS levels.

### 2.5. Hoechst 33258 Staining

The nuclear fragmentation in MCF-7 cells treated with different concentrations of GAL (0, 0.5, 1, and 2 *μ*mol/L) for 24 h was visualized using Hoechst 33258 staining. MCF-7 cells were plated in 6-well plates at a density of 5 × 10^4^ cells per well and incubated with GAL. After 24 h, the cells were incubated with Hoechst 33258 (5 *μ*g/mL) for 30 min at room temperature. Following washing with PBS, the cells were visualized and photographed under an Olympus inverted fluorescence microscope (Tokyo, Japan).

### 2.6. Flow Cytometric Analysis of Cell Apoptosis

Apoptosis was determined using an annexin V-FITC apoptosis kit (BD Pharmingen, Franklin Lakes, NJ, USA) according to manufacturer's instructions. After treatment with GAL (0, 0.5, 1, and 2 *μ*mol/L) for 24 h, cells were washed with ice-cold PBS and resuspended in binding buffer (10 mmol/L HEPES, pH 7.4, 140 mmol/L NaCl, and 2.5 mmol/L CaCl_2_) at a concentration of 1 × 10^6^ cells/mL. Cells were stained with annexin V-FITC and propidium (PI) for 15 min in dark before being analyzed with flow cytometer (Beckman Coulter Inc., Miami, FL, USA).

### 2.7. Western Blotting Analysis

MCF-7 cells were treated with various concentrations of GAL (0, 0.25, 0.5, 1, and 2 *μ*mol/L) for 24 h in 25 cm^2^ flask. The cells were collected, washed twice with PBS, and then lysed with RIPA buffer and protease and phosphatase inhibitors cocktail (Roche, Beijing, China) for 20 min on ice. The cell lysates were cleared by centrifugation at 12,000 g for 20 min. Protein concentrations were determined by Bradford assay. Equal amounts of lysate (40 *μ*g) were resolved by SDS-PAGE and transferred to polyvinylidene difluoride membrane (Millipore Corp., Bedford, MA, USA). Membranes were blocked in TBST containing 5% nonfat skim milk at room temperature for 2 h and probed with primary antibodies overnight at 4°C. Then membranes were blotted with an appropriate horseradish peroxidase-linked secondary antibody. Proteins were visualized using enhanced chemiluminescence Western blotting detection reagents (Amersham Pharmacia Biotech, Inc., Piscataway, NJ, USA).

### 2.8. RNA Interference

FOXO3a (FKHRL1) small interfering RNA (siRNA) and the scrambled siRNA were purchased from Santa Cruz Technology (Dallas, TX, USA). After FOXO3a siRNA or NC siRNA was transfected into MCF-7 cells using silMPORTER (Upstate, Virginia, USA) for 24 h, 1 *μ*mol/L GAL was added for 24 h. After GAL treatment for 24 h, the cells were harvested. Then, cells apoptosis was checked through Hoechst staining and protein expression correlated with apoptosis was detected by Western blotting analysis.

### 2.9. Statistical Analysis

Data are presented as mean ± standard deviation (SD). Statistical analysis was performed using SPSS 11.5 software (SPSS Inc., Chicago, Illinois, USA). Comparison between groups was performed with Student's *t*-test. A *P* value of ≤0.05 was considered statistically significant.

## 3. Results

### 3.1. The Molecular Structure and the Identification of GAL

The molecular weight of GAL is 775 and molecular formula is C_44_H_58_N_2_O_10_ (Figure 1(a)). GAL is determined by Matrix-Assisted Laser Desorption/Ionization Time of Flight Mass Spectrometry and the peak value is 755.4 Da (Figure 1(b)).

### 3.2. GAL Inhibited the Proliferation of MCF-7 Cells and NAC Could Block Its Inhibition

**Figure 2:**
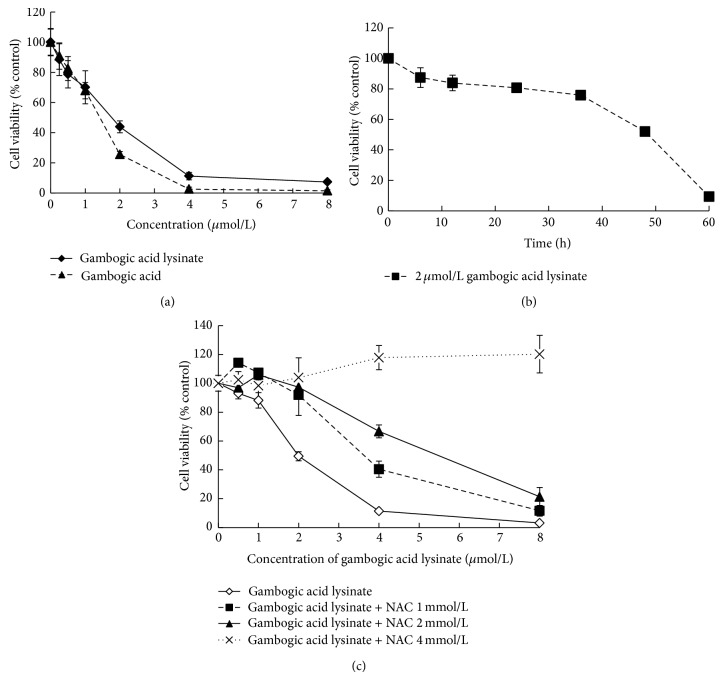
Gambogic acid lysinate can inhibit MCF-7 cell proliferation; however NAC can block it. (a) Exponential growth MCF-7 cells were treated with various concentrations of gambogic acid lysinate (0, 0.25, 0.5, 1, 2, 4, and 8*μ*mol/L) for 48 h; (b) exponential growth MCF-7 cells were treated with 2*μ*mol/L gambogic acid lysinate for different times (0, 6, 12, 24, 36, 48, and 60 h); (c) exponential growth MCF-7 cells were treated with various concentrations of gambogic acid lysinate (0, 0.25, 0.5, 1, 2, 4, and 8*μ*mol/L) plus various concentrations of NAC (0, 1, 2, and 4 mmol/L) for 48 h, and cell viability was detected by MTT method.

The proliferation inhibitory effect of GAL in human MCF-7 cells was examined with MTT assay as described in Section 2. Cells were cultured for 48 h (Figure 2(a)) in the presence of various concentrations of GAL. MCF-7 cells showed a decreased cell proliferation after treatment with GAL. The IC_50_ value of GAL for MCF-7 cells is 1.46 *μ*mol/L comparable with GA (IC_50_, 1.16 *μ*mol/L). Cells were cultured for different times (0, 6, 12, 24, 36, 48, and 60 h) in the presence of 2 *μ*mol/L GAL. MCF-7 cells showed a decreased cell proliferation over time (Figure 2(b)). In addition, the proliferation inhibitory effect of GAL in human MCF-7 cells was blocked, when NAC (0, 1, 2, and 4 mmol/L) was added in combination with GAL (Figure 2(c)).

### 3.3. Induction of Oxidative Stress in MCF-7 Cells

**Figure 3:**
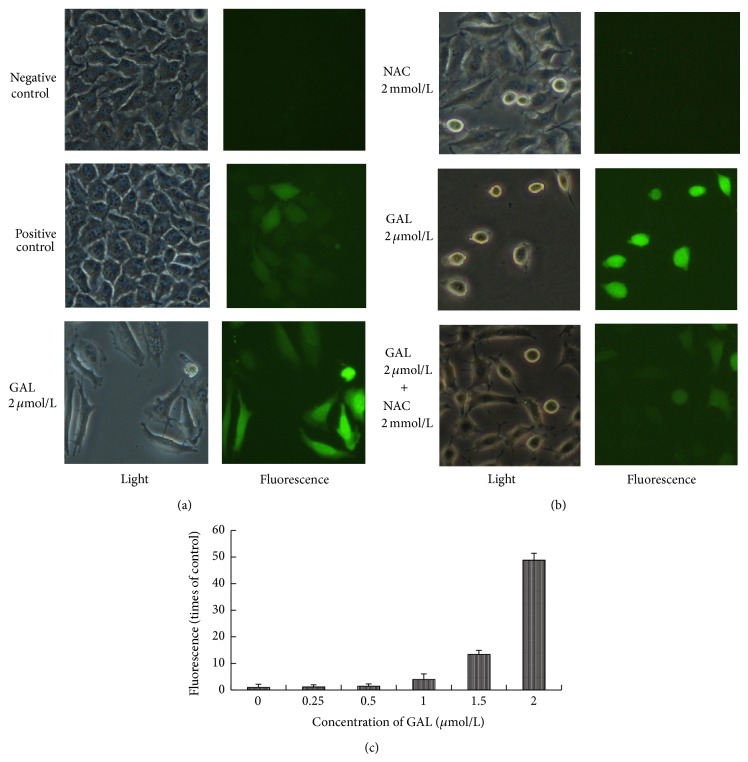
Gambogic acid lysinate can increase intracellular ROS level and NAC can block it. Exponential growth MCF-7 cells were treated with PBS (negative control), Rosup 100 mg/L (positive control), gambogic acid lysinate 2*μ*mol/L, NAC 2 mmol/L, and gambogic acid lysinate 2*μ*mol/L + NAC 2 mmol/L for 24 h. After treatment MCF-7 cells were stained with DCFH-DA and were determined by fluorescence microscopy (a, b) and fluorescence spectrophotometer (c).

The intracellular ROS level was stained with DCFH-DA and determined by fluorescence microscopy and fluorescence spectrophotometer as described in Section 2. GAL could increase ROS level in dose dependent manner; however NAC could decrease the increase of ROS induced by GAL (Figure 3).

### 3.4. Induction of Apoptosis by GAL in MCF-7 Cells

**Figure 4:**
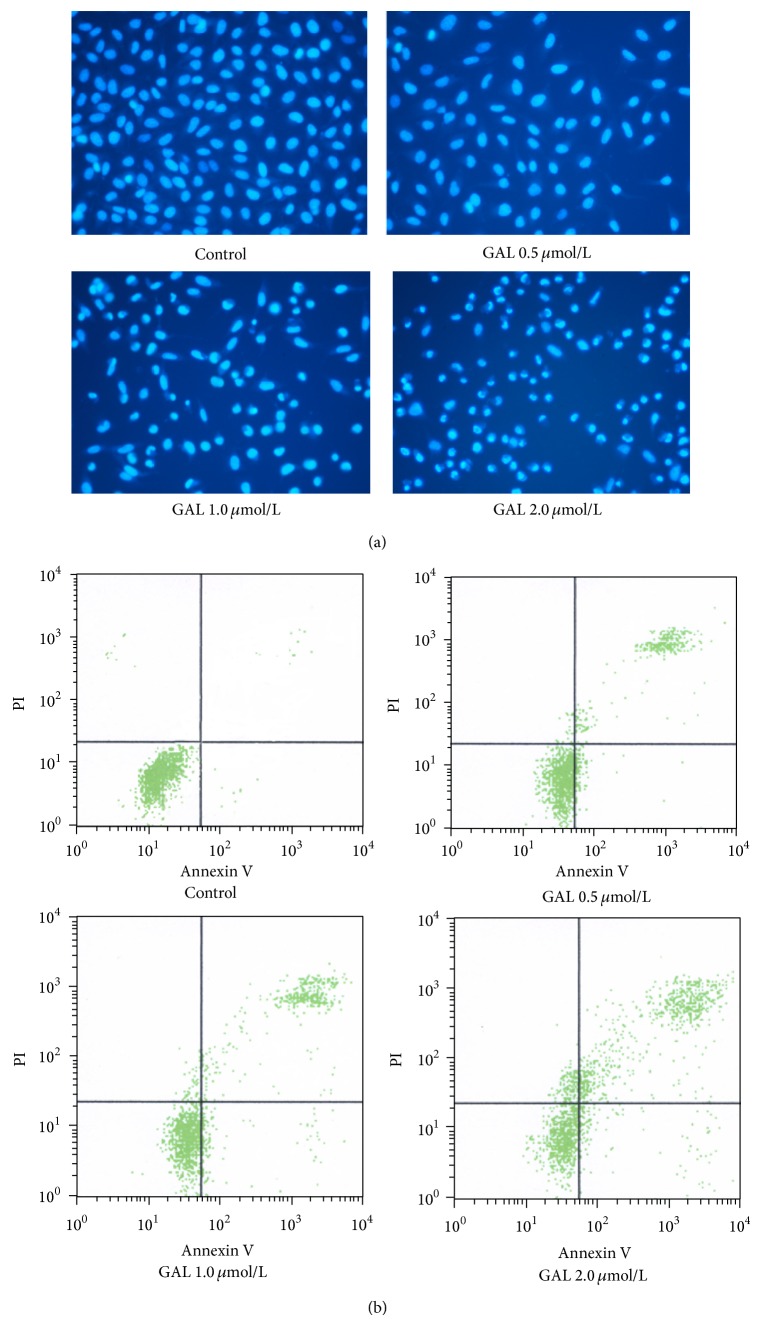
Gambogic acid lysinate can induce MCF-7 cell apoptosis. Exponential growth MCF-7 cells were treated with GAL (0, 0.5, 1, 1.5, and 2*μ*mol/L) for 24 h. (a) After treatment MCF-7 cells were stained with Hoechst 33258 and observed by fluorescence microscope. (b) After treatment MCF-7 cells were stained with annexin V-FITC and PI and analyzed with flow cytometer.

By Hoechst 33258 staining the nuclei of untreated cells were normal in appearance and showed diffused staining of the chromatin. After exposure to GAL for 24 h, most cells presented typical morphological changes of apoptosis such as chromatin condensation, cell shrinkage, chromatin margination, or apoptotic bodies (Figure 4(a)). Induction of apoptosis by GAL was further confirmed by annexin V-FITC/PI staining. GAL (0.5, 1, 2 *μ*mol/L) induced apoptosis in MCF-7 cells in dose dependent manner and the ratio of apoptosis was 23.07%, 28.9%, and 43.25%, respectively (Figure 4(b), Table 1). It suggested that apoptosis was the predominant mode of GAL-induced cell death.

### 3.5. Downregulation of SIRT1 and Upregulation of C-Caspase-3 in GAL-Induced Apoptosis in MCF-7 Cells

**Figure 5:**
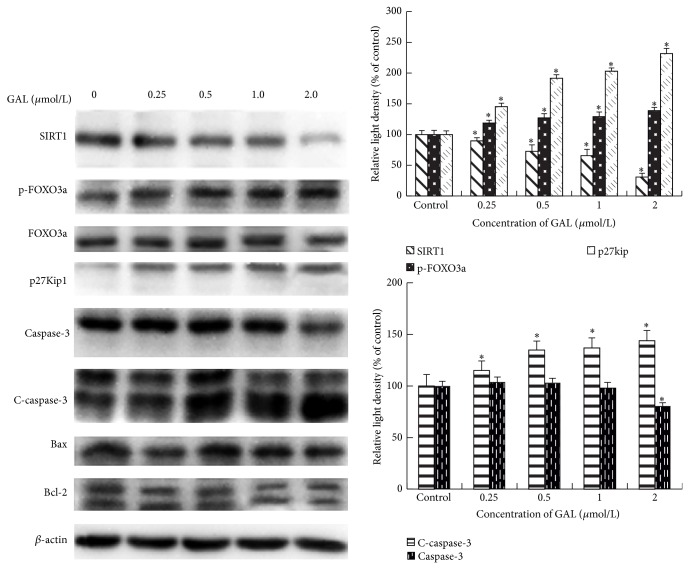
Gambogic acid lysinate can induce MCF-7 cells apoptosis by SIRT1 and caspase-3 signal pathway. MCF-7 cells were treated with the indicated concentrations of GAL for 24 h, and the cell extracts were prepared and analyzed by Western blotting with corresponding antibodies. The blots were quantified by densitometry with Scion Image (Scion Corporation, Frederick, MD, USA), and the relative ratio of target protein to*β*-actin was calculated and expressed as the mean ± SD from three experiments. ^*^ *P* < 0.05 versus the control group.

In order to investigate the role of apoptosis, the activity of SIRT1 and caspase-3 in response to GAL treatment was determined. SIRT1 expression was decreased by GAL in a dose dependent manner. However, the phosphorylation of FOXO3a and the expression p27Kip1 were increased by GAL in a dose dependent manner. In addition the expression of cleaved caspase-3 also increased in a dose dependent manner; however the expression of caspase-3 decreased at GAL 2 *μ*mol/L treated group (Figure 5). There is no change of the expression of Bcl-2 and Bax.

### 3.6. FOXO3a siRNA Partly Blocked Cell Apoptosis Induced by GAL in MCF-7 Cells

**Figure 6:**
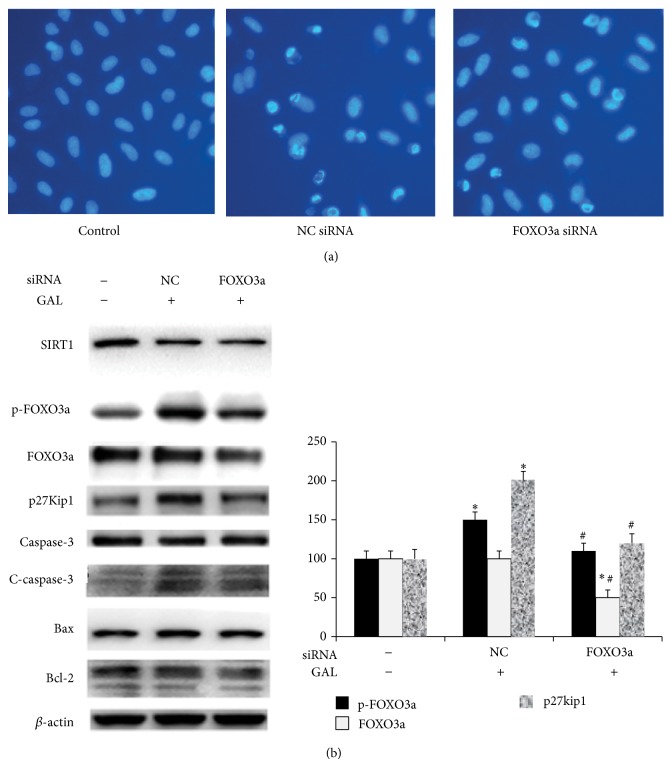
FOXO3a siRNA partly blocked cell apoptosis induced by GAL in MCF-7 cells. MCF-7 cells were transfected with NC or FOXO3a siRNA for 24 h; then 1*μ*mol/L GAL was added for 24 h. After GAL treatment for 24 h, (a) MCF-7 cells were stained with Hoechst 33258 and observed by fluorescence microscope; (b) MCF-7 cells were harvested and cell extracts were prepared and analyzed by Western blotting with corresponding antibodies. The blots were quantified by densitometry with Scion Image (Scion Corporation, Frederick, MD, USA), and the relative ratio of target protein to*β*-actin was calculated and expressed as the mean ± SD from three experiments. ^*^ *P* < 0.05 versus the control group; ^#^ *P* < 0.05 versus the GAL+NC siRNA group.

Compared with control group, after exposure to GAL+NC siRNA for 24 h, most cells presented typical morphological changes of apoptosis such as chromatin condensation, cell shrinkage, chromatin margination, or apoptotic bodies. Compared with GAL+NC siRNA group, GAL+FOXO3a siRNA group partly blocked the effect of GAL (Figure 6(a)). By Western blot analysis we found that FOXO3a siRNA decreased the expression of FOXO3, the phosphorylation of FOXO3, and the expression of p27Kip1, but with no effect on the expression of SIRT1, caspase-3, Bax, and Bcl-2 (Figure 6(b)).

## 4. Discussion and Conclusions

Breast cancer is the most common cancer among women in China and western countries. It is also the principal cause of cancer death for females. As a result of “westernized lifestyles” and exogenous estrogen exposure, there is an increasing trend of breast cancer incidence in China in the latest decades [1, 12]. SIRT1 physiologically interacts with p53 and attenuates its functions through deacetylation at its C-terminal Lys382 residue [13]. The expression of SIRT1 protein was seen in most human breast cancer specimens, and its expression was significantly associated with distant metastasis and poor prognosis [14–16] and reported to function as a tumor promoter. Recently, SIRT1 has emerged as a potent therapeutic target to cancer treatment. Therefore, identification of potent and unique SIRT1 inhibitor for cancer treatment is urgently needed.

GAL is the salt of lysine and gambogic acid, which has antitumor activities in a broad range of human cancer cells [17]. The difference between GAL and GA is that GAL can dissolve in water; however GA cannot. In this study, we observed that the inhibition of GAL to MCF-7 cell proliferation was comparable to that of GA and the IC_50_ values of GAL and GA were 1.46 and 1.16 *μ*mol/L, respectively. GAL could inhibit MCF-7 cell proliferation in dose and time dependent manner (Figures 2(a) and 2(b)). We also found that NAC can block the activity of GAL, when GAL was administrated in combination with NAC (1, 2, and 4 mmol/L). The block of NAC to the activity of GAL was increased with the increase of the concentration of NAC (Figure 2(c)). It is well known that NAC is an ROS scavenger and is frequently used as a precursor to GSH, which supports the synthesis of GSH, and assists in replenishing GSH when stores are compromised during oxidative or electrophilic stress [18, 19]. It can be deduced that GAL can inhibit MCF-7 cell proliferation by increasing ROS level, and NAC can remove the ROS induced by GAL. It is further confirmed by fluorescence microscope and fluorescence spectrophotometer by which intracellular ROS can be determined.

It is well known that ROS can induce cell apoptosis by various signal pathways including targeting cytosolic thioredoxin reductase [20], JNK/ATF2 pathway [21], p53-mitochondrial pathway [22], and AMPK/SIRT1/PGC-1*α* signal pathway [23]. It has been reported that GA has the ability to activate apoptotic signaling in numerous types of cancer cells. Herein, we also demonstrated that GAL kills MCF-7 cells predominantly through induction of apoptosis (Figure 4). GAL triggers apoptotic cell death in dose dependent manner. In Western blot analysis we also observed that SIRT1 expression was decreased in dose dependent manner. SIRT1 is one of the cytoplasmic NAD^+^-dependent histone deacetylases and deacetylates histone H3 lysine 56 (H3K56) and a-tubulin. It also shares nonhistone substrates of FOXO1, FOXO3, and p53 with SIRT1 [24]. In previous study it is reported that forkhead transcription factors can be inhibited by the deacetylase SIRT1 [25], and increased expression of any FOXO member results in the activation of the cell cycle inhibitor p27Kip1 [26]. In this study we investigated the change of FOXO3a and p27Kip1. We found that the phosphorylation of FOXO3a and the expression of p27Kip1 increased with the decrease of SIRT1 expression. It can be concluded that SIRT1/FOXO3a/p27Kip1 signal pathway is one of the pathways by which GAL induced MCF-7 cell apoptosis. In the meantime we also observed that GAL enhanced the expression of cleaved caspase-3. It can be inferred that caspase-3 signal pathway also takes part in the apoptotic process induced by GAL. At knockdown of FOXO3a, we found the expression of FOXO3, the phosphorylation of FOXO3, and the expression of p27Kip1 were decreased and cell apoptosis induced by GAL was partly blocked; however the expression of SIRT1, caspase-3, Bax, and Bcl-2 did not change (Figure 6(b)). It was further confirmed that SIRT1/FOXO3a/p27Kip1 signal pathway is only one of the pathways by which GAL induced MCF-7 cell apoptosis.

In conclusion these results suggested that GAL inhibited MCF-7 cells proliferation and induced MCF-7 cells apoptosis by increasing ROS level which could induce cell apoptosis by both SIRT1/FOXO3a/p27Kip1 and caspase-3 signal pathway. GAL may be a new chemotherapy drug for breast cancer.

## Figures and Tables

**Table 1:** The apoptosis ratio of different groups.

Groups	Normal (%)	Necrosis (%)	Early apoptosis (%)	Late apoptosis (%)
Control	96.20	0.50	1.00	2.30
GAL 0.5 *µ*mol/L	75.84	1.09	5.45	17.62
GAL 1.0 *µ*mol/L	68.92	2.17	5.95	22.95
GAL 2.0 *µ*mol/L	47.62	9.14	5.90	37.35
